# 71016 Defining "rurality": Rural-urban disparities among COPD patients in national VA data

**DOI:** 10.1017/cts.2021.747

**Published:** 2021-03-30

**Authors:** Arianne Baldomero, Ken M. Kunisaki, Patrick Hammett, David Nelson, Carrie Henning-Smith, Ann Bangerter, Chris H. Wendt, R. Adams Dudley

**Affiliations:** 1 Minneapolis VA Health Care System; 2 University of Minnesota; 3 University of Minnesota

## Abstract

ABSTRACT IMPACT: Our research focuses on determining rural-urban disparities in chronic obstructive pulmonary disease (COPD) management to improve COPD health outcomes in rural areas. OBJECTIVES/GOALS: Several methods exist to distinguish rural from urban areas, but it is not clear which method relates most directly to rural-urban health care disparities. To address this, we compared different measures of rurality to measures of chronic obstructive pulmonary disease (COPD) processes of care among a national sample of veterans. METHODS/STUDY POPULATION: Retrospective analysis of patients with COPD (2016-2019 by ICD-10 codes) using national Veterans Affairs (VA) data. We assessed rurality by: 1) patient’s residential address, 2) assigned primary care clinic address, and 3) drive time from the patient’s residence to closest primary care clinic. Rurality designations of the residential address and primary care clinic address into urban, rural, and highly rural areas are based on the Rural Urban Commuting Area (RUCA) codes. The dependent variables were binary outcomes of: 1) documentation of a pulmonary clinic encounter and 2) evidence of spirometry to confirm the diagnosis of COPD. RESULTS/ANTICIPATED RESULTS: Of 6,765,951 veterans, 1,157,002 (17%) had COPD (Table 1). Although approximately 40% of patients with COPD reside in addresses that are rural and highly rural, a large majority are assigned to primary care clinics in urban areas (82.8%) and reside within 30 minutes to the closest primary care clinic (76.7%) (Table 2). Compared to defining rurality based on patient’s residential address or drive time to closest primary care, defining rurality based on the assigned primary care clinic address was associated with a larger disparity in rates of pulmonary encounter. In contrast, the drive time from the patient’s residence to the closest primary care was the strongest predictor of receipt of spirometry (Figure 1 and Table 3). DISCUSSION/SIGNIFICANCE OF FINDINGS: Estimates of the severity of rural-urban disparities varied based on the definition of rurality used. For two process measures, definitions of rurality based on where the patient received primary care generated more evidence of disparities than definitions based solely on the patient’s residential address.

